# Synthesis, characterization, and Hirshfeld surface analysis of coordination compounds composed of two different aminopyridines, isothiocyanate ligand molecules, and two different transition metal atoms

**DOI:** 10.55730/1300-0527.3697

**Published:** 2024-09-17

**Authors:** Zarife Sibel ŞAHİN, Zeki KARTAL

**Affiliations:** 1 Department of Energy Systems Engineering, Faculty of Engineering and Architecture, Sinop University, Sinop, Turkiye; 2 Retired Professor of Atomic and Molecular Physics, Kütahya Dumlupınar University, Kütahya, Turkiye

**Keywords:** 3-Aminopyridine (3AP), single crystal X-ray diffraction analysis, 4-aminopyridine (4AP), Fourier transform infrared spectroscopy, density functional theory, Hirshfeld surfaces

## Abstract

In this article, we describe the successful synthesis of three coordination compounds formed by the ligands 3-aminopyridine, 4-aminopyridine, and isothiocyanate ion with copper atoms and 4-aminopyridine and isothiocyanate ion with cadmium atoms, and their structural characterizations. The crystal structures of the compounds were determined by single crystal X-ray diffraction. According to that technique, the open formulae of these compounds are [Cu(3-aminopyridine)_2_(NCS)_2_] (**1**), [Cu(4-aminopyridine)_3_(NCS)_2_] (**2**), and [Cd(4-aminopyridine)_2_(NCS)Cl] (**3**). In addition, the suitability of the structures of the compounds was characterized by elemental analysis, thermal analysis, and Fourier transform infrared spectroscopy. The single crystal X-ray diffraction analyses of these coordination compounds showed that the first of these coordination compounds had a 1D crystal structure and the other two had a 3D crystal structure. N-H⋯S, N-H⋯N, N-H⋯Cl, N-H⋯π, and C-H⋯π bonds and their combinations were effective in the formation of the crystal structures of the said coordination compounds. The metal atoms [Cu(II), Cu(II), and Cd(II)] in these coordination compounds were surrounded by various ligand molecules in a square planar, square pyramidal, and octahedral arrangement, respectively. In order to investigate some chemical and structural properties of these coordination compounds, theoretical calculations were performed with the software package Gaussian 03. The highest occupied molecular orbital (HOMO), lowest occupied molecular orbital (LUMO), and natural bond orbital (NBO) values of the coordination compounds were used in these calculations. When the energy gap value between the HOMO and LUMO states of the compounds was examined, it was predicted that compound **3** may have lower kinetic stability, higher chemical activity, and lower semiconductor properties than all the other compounds. According to the Hirshfeld surface analysis of the compounds, C⋯H, S⋯H, H⋯H, and N⋯H interactions are generally seen in the crystal structures of all compounds. In addition, Cd⋯Cl, Cd⋯S, H⋯Cl, and Cl⋯Cl interactions also occur in compound **3**.

## 1. Introduction

In general, one of the most important properties of metal atoms is that they can form compounds with a large number of Lewis bases by acting as Lewis acids. Metal complexes are also often referred to as “coordination compounds”. Coordination compounds consist of a central metal atom and neutral and charged ligand molecules bound to it according to certain rules [1–3]. In a coordination compound, coordinate bonds are formed between ligands that donate their unpaired electrons and metal atoms or metal ions that receive these unpaired electrons. The number of electron pairs shared with the metal atom or metal ion in a coordination compound is known as the “coordination number” of that compound. The coordination numbers in various coordination compounds can range from 1 to 8. However, the most common coordination numbers observed in various coordination compounds are 2, 4, and 6. In order for unpaired electrons in a ligand molecule to be shared with a metal atom or ion, there must be an empty orbital on the metal atom or an ion that can accept these unpaired electrons. The spatial orientation of these empty orbitals on the metal atom or ion determines the shape of the coordination compound that will occur in 3D space (for example, square planar, octahedral, and tetrahedral) [3,4]. The structures of these coordination compounds in question can vary from a very simple form to a more complicated one. This enormous variability in the structures of coordination compounds makes them complexes of great importance both naturally and industrially. Coordination compounds with positive or negative electrical charges are also called “complex ions”. A coordination compound can often contain one or more complex ions [1–5].

Since most of the elements that make up the periodic table are metals and almost all metals can form complexes, many important catalysts in industry are metal complexes, and transition metal complexes are of great biochemical importance, interest in coordination compounds in scientific studies is increasing [2,3,5]. In 1893, Alfred Werner proposed a theory named after him to explain the existence and structure of coordination compounds [6]. The coordination compounds are divided into various groups and named according to their properties. Among these compound groups, Hofmann-type and Werner-type compounds are the most important [1,2].

The closed formula of some coordination compounds synthesized by Alfred Werner is given as [MX_2_A_4_]. In this formula, M is a divalent cation [M(II) = Cr(II), Mn(II), Fe(II), Co(II), Ni(II), Cu(II), Zn(II), Cd(II), and Hg(II)], X is an anionic ligand (X = NCS^−^, NCO^−^, CN^−^, NO_3_^−^, Cl^−^, Br^−^, I^−^), and A is a neutral ligand. This neutral ligand is usually pyridine, pyridine derivatives, and compounds such as amine or quinoline and others [6–8].

Compounds known as aminopyridines (APs), with the closed chemical formula C_5_H_6_N_2_, consist of an amino group in ortho, meta, and para states relative to the nitrogen atom in a pyridine ring. Aminopyridines are called 2-aminopyridine, 3-aminopyridine, and 4-aminopyridine and are briefly shown as 2AP, 3AP, and 4AP, respectively. All APs and their derivatives are used both in the treatment of various diseases in the field of medicine and in the production of many new chemical compounds [9–12].

The copper atom has a feature that plays an important role in many biological events such as respiration, energy metabolism, and DNA synthesis and in chemical synthesis events for obtaining many new coordination compounds [13]. Compounds containing copper atoms in their structure are becoming the focus of increasing attention due to their different magnetic and electrical properties, therapeutic properties against many diseases, and many other structural properties [14].

The cadmium(II) ion has similar behavior to the copper(II) ion in terms of coordination properties. First of all, the behavior of the Cd(II) ion in compound formation is in the form of a soft Lewis acid. Therefore, the Cd(II) ion forms stable complexes with ligands containing S^2−^ and HS^−^ groups. In addition, there are many compounds formed with ligands containing oxygen donor groups. The Cd atom has a wide coordination ability that can produce compounds with various structures, from very simple complexes to 3D polymeric compounds. Polymeric structures formed with the help of Cd atom have very interesting physical properties. In addition, the Cd(II) ion can replace Zn(II) ions in some enzymes. Due to these and similar properties, the coordination chemistry of the Cd atom has been the most frequently researched area in recent years [15,16].

Our previous studies were related to metal chloride, metal cyanide, Hofmann-type and Hofmann-type-like compounds, and clathrates obtained with various aminopyridine ligands [17–21]. Our next studies will be about Werner-type and Werner-type-like compounds and clathrates formed by various aminopyridines and isothiocyanate ligands with different transition metals.

Our current work is about Werner-type-like compounds. The crystal structures of these compounds do not exactly fit the formula of Werner-type compounds. The number of ligands in them and the 3D positions of these ligand molecules in the compound may differ. The aim of the present study was to form new Werner-type-like compounds consisting of copper and cadmium transition metal atoms in crystal form by using 3AP and 4AP molecules and an isothiocyanate (NCS)^−^ ion as ligand. The isothiocyanate (NCS)^−^ anion is a monovalent basic anion with a linear triatomic structure. The isothiocyanate (NCS)^−^ anion can form various kinds of chemical bonds from a carbon atom, sulfur atom, nitrogen atom, or π electrons in chemical reactions [22,23].

The chemicals 3AP, 4AP, K(NCS), CuCl_2_, and CdCl_2_ were used in different ratios to obtain the crystal forms of Werner-like-type compounds. As a result of that study, three different Werner-like-type compounds ([Cu(C_5_H_6_N_2_)_2_(NCS)_2_], [Cu(C_5_H_6_N_2_)_3_(NCS)_2_], and [Cd(C_5_H_6_N_2_)_2_(NCS)Cl]) were obtained in crystal form. In the present study, information was given about the spectroscopic and some electronic properties of Werner-like-type compounds with three different structures obtained in crystal form. In addition, some calculations were performed with the software package Gaussian 03 to calculate some of the electrical and thermal properties of these three new compounds. An important issue is the presence of the properties of storing guest molecules in the obtained compounds and the comparison of these properties with those of other compounds. For this reason, the program CrystalExplorer was used to calculate the voids in the structures of the compounds. In addition, all interactions involved in the formation of the compounds, their charge distributions, and energies were calculated with the help of CrystalExplorer.

The aim of the present study was to obtain new Werner-type-like compounds in crystal form using Cu(II) and Cd(II) transition metals and 3AP, 4AP, and (NCS)^−^ ligands and to calculate some of their properties. The effects of changing the ligand molecule, the numbers of the same ligand molecule, and the changing of the transition metal atom on the changes in various properties of the mentioned compounds were investigated in Werner-type-like compounds.

## 2. Experimental

### 2.1. Materials

In order to obtain the targeted compounds, the following chemicals were used: 3AP, 4AP (C_5_H_6_N_2_), (Sigma Aldrich, 99%); potassium thiocyanate (KSCN) (Fluka, 96%); anhydrous copper(II) chloride (CuCl_2_) (Fluka, 99%); cadmium(II) chloride monohydrate [CdCl_2_·H_2_O], (Sigma Aldrich, 99+%), and ammonia solution (NH_3_, Merck, 25%).

### 2.2. Syntheses of compounds

The following chemical processes were applied to obtain compounds in crystalline structure.

For compound **1**, 2 mmol of KSCN (0.196 g) was dissolved in 10 mL of distilled water with continuous stirring, and 2 mmol of 3AP (0.188 g) was added to this solution. Then 1 mmol of anhydrous CuCl_2_ (0.135 g) was dissolved in 5 mL of distilled water and added to the previous mixture, and the entire mixture was stirred at a constant temperature of 60 °C for 20 min. Then the aqueous ammonia solution was added dropwise to this mixture and a clear image of the whole mixture was obtained. This clear-looking mixture was stirred for another 20 min at a constant temperature of 60 °C, then filtered, and allowed to crystallize under normal room conditions. Light blue, transparent crystals of this compound formed within about 4 weeks.

For compound **2**, 2 mmol of KSCN (0.196 g) was dissolved in 10 mL of distilled water with continuous stirring, and 3 mmol of 4AP (0.282 g) was added to this solution. Then 1 mmol of anhydrous CuCl_2_ was dissolved in 5 mL of distilled water and added to the previous mixture, and the entire mixture was stirred at a constant temperature of 60 °C for 20 min. Then the aqueous ammonia solution was added dropwise to this mixture and a clear image of the whole mixture was obtained. This clear-looking mixture was stirred for another 20 min at a constant temperature of 60 °C, then filtered, and allowed to crystallize under normal room conditions. Blue, transparent crystals of this compound formed within about 5 weeks.

For compound **3**, 2 mmol of KSCN (0.196 g) was dissolved in 10 mL of distilled water with continuous stirring, and 2 mmol of 4AP (0.188 g) was added to this solution. Then 1 mmol of CdCl_2_.H_2_O (0.202 g) was dissolved in 5 mL of distilled water and added to the previous mixture and the entire mixture was stirred at a constant temperature of 60 °C for 20 min. Then the aqueous ammonia solution was added dropwise to this mixture and a clear image of the whole mixture was obtained. This clear-looking mixture was stirred for another 20 min at a constant temperature of 60 °C, then filtered, and allowed to crystallize under normal room conditions. Colorless, transparent crystals of this compound formed within about 2 weeks.

### 2.3. Instrumentation

Fourier transform infrared (FTIR) spectra of all crystalline compounds were recorded using a Bruker Optics Vertex 70 FTIR spectrometer. The metal amounts and carbon, nitrogen, and hydrogen amounts in all the compounds obtained were analyzed by PerkinElmer Optima 4300 DV ICP-OES device and CHNS-932 (LECO) elemental measuring device, respectively. Data on the crystal structures of all three compounds were collected with a D8-QUEST diffractometer (Bruker Optics) equipped with graphite monochromatic Mo-*K*_α_ (λ = 0.71073 Å) radiation. Thermogravimetric analysis (TGA), differential thermogravimetry (DTG), and differential thermal analysis (DTA) curves of the compounds were recorded using an SII EXSTAR 6000 TG/DTA 6300 model thermal analyzer using platinum crucibles at a heating rate of 5 °C/min in nitrogen environment in the 30–950 °C temperature range.

Experimentally measured and theoretically calculated values of all elements in the structures of all compounds in crystalline form are given in Table S1. The theoretical and experimental values of the elements in the structure of the compounds are in good agreement with each other.

Appropriate crystals of each compound were selected for data collection performed on a Bruker diffractometer with graphite-monochromatic Mo-*K*_α_ radiation at 296 K. The following procedures were used for our analyses: resolved by direct methods: SHELXS-2013 [24]; refined by full matrix least squares methods: SHELXL-2013 [25]; data collection: Bruker APEX2; molecular graphics: mercury [26]; solution: WinGX [27]. Details of the data collection and crystal structure determination are given in Table S2 for all compounds.

Regarding the structures of the three compounds obtained in crystalline form, it was understood from their single crystal X-ray diffusion (SC-XRD) data and elemental analysis that they were Werner-type-like compounds, and their closed formulas could be given as [Cu(3-aminopyridine)_2_(NCS)_2_], [Cu(4-aminopyridine)_3_(NCS)_2_], and [Cd(4-aminopyridine)_2_(NCS)Cl]. Hereinafter these compounds will be denoted as [Cu(3-aminopyridine)_2_(NCS)_2_] (**1**), [Cu(4-aminopyridine)_3_(NCS)_2_] (**2**), and [Cd(4-aminopyridine)_2_(NCS)Cl] (**3**) for short.

The atomic numbering schemes and molecular structures of the compounds obtained in crystalline form, according to the SC-XRD experimental data, are given in the text.

It is seen that the first two compounds (**1** and **2**) synthesized in crystal form were obtained in accordance with the synthesis purposes. However, it is seen that there is a chlorine atom in the structure of the third compound (**3**) instead of an isothiocyanate ligand, separate from its synthesizing purpose.

## 3. Results and discussion

### 3.1. Crystallographic analyses of compounds 1–3

#### 3.1.1. Crystallographic analysis of compound 1

**Figure 1:**
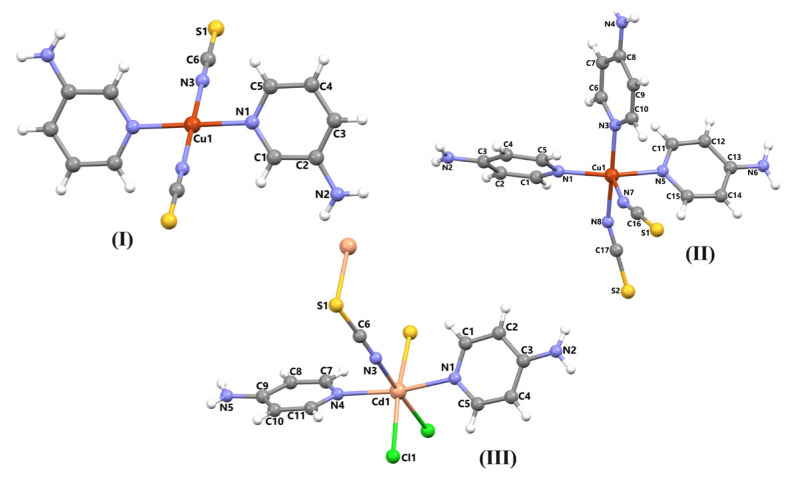
The molecular structures of the compounds showing the atom numbering scheme.

The molecular structure of compound **1**, with the atom numbering scheme, is shown in Figure 1. The asymmetric unit of compound **1** consists of half a Cu(II) ion, one 3AP ligand, and one isothiocyanate molecule. The Cu(II) ion is coordinated by two nitrogen atoms [Cu1-N1 = 2.013(6) Å] from 3AP ligands and two nitrogen atoms [Cu1-N3 = 1.963(7) Å] from isothiocyanate molecules, thus showing a square planar coordination geometry. The bond distances and bond angles between some atoms in the investigated compounds **1**, **2**, and **3** are given in Table 1. The molecules of **1** are linked into sheets by a combination of N-H⋯S hydrogen bonds (Table S3).

**Figure 2:**
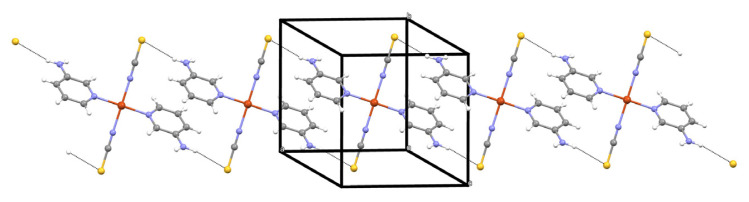
An infinite 1D layer in **1**.

The N2 atom of the amino group acts as hydrogen-bond donor to atom S1 in the molecule at (2 – x, 1 – y, 1 – z), forming a centrosymmetric R_2_^2^(18) ring centered at (n + 1, 1/2, 1/2) (n = zero or integer) (Figure 2). Compound **1** also contains one π⋯π interaction, which occurs between the two symmetry-related 3AP ligands. The distance between the centers of the two interacting pyridine rings is 3.5679(4) Å.

#### 3.1.2. Crystallographic analysis of compound 2

The asymmetric unit of compound **2** consists of one Cu(II) ion, three 4AP ligands, and two isothiocyanate molecules (Figure 1). The Cu(II) ion is coordinated by three nitrogen atoms [Cu1-N1 = 2.006(6) Å, Cu1-N3 = 2.018(5) Å and Cu1-N5 = 2.028(5) Å] from 4AP ligands and two nitrogen atoms [Cu1-N7 = 2.175(7) Å and Cu1-N8 = 2.018(6) Å] from isothiocyanate molecules, thus showing a square pyramidal coordination geometry. The N2 atom of the amino group acts as a hydrogen-bond donor to atoms S1 and S2 in the molecule at (x + 1, y, z), forming a C(10) chain running parallel to the [100] direction. These chains produce R_2_^2^(10) rings. Similarly, the amino N4 atom acts as a hydrogen-bond donor to atom S2 in the molecule at (1 – x, y – 1/2, 3/2 – z), forming a C(10) zigzag chain running parallel to the [010] direction.

**Figure 3:**
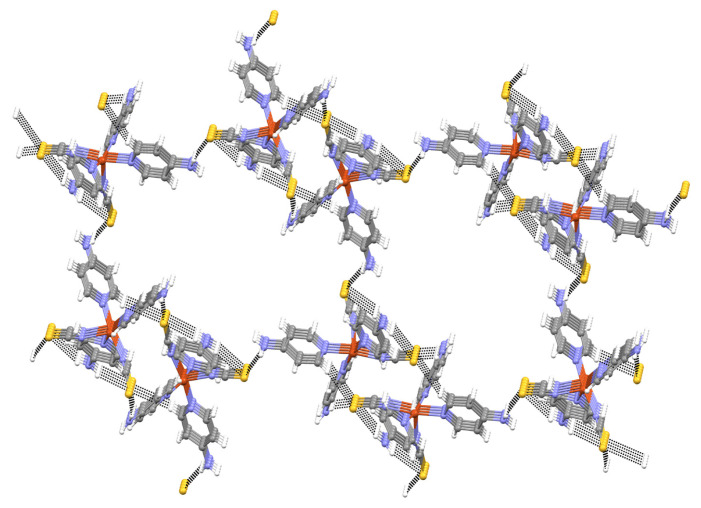
An infinite 2D layer in **2**.

Compound **2** also contains one N-H⋯π interaction. The amino N4 atom in the molecule at (x, y, z) acts as a hydrogen-bond donor to the C11-C15/N5 pyridine ring in the molecule at (x + 1/2, y, 3/2 – z), thus forming a C(9) chain running parallel to the [100] direction. The combination of these interactions creates a 3D network in the crystal structure of compound **2** (Figure 3).

#### 3.1.3. Crystallographic analysis of compound 3

The SC-XRD study shows that compound **3** is a 1D coordination polymer. The asymmetric unit of compound **3** consists of one Cd(II) ion, two 4AP ligands, one isothiocyanate molecule, and one coordinated chlorine atom (Figure 1).

**Figure 4:**
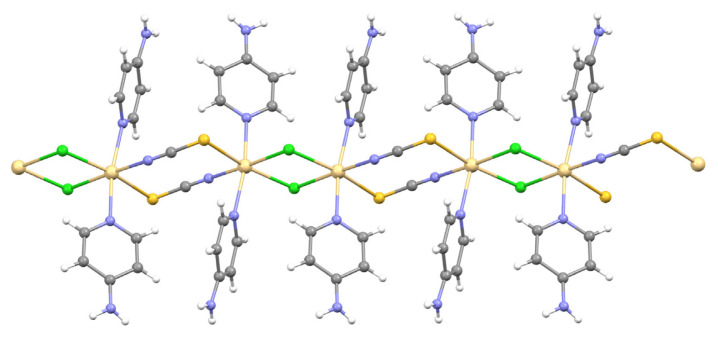
An infinite 1D layer in **3**.

The Cd(II) ion is coordinated by two nitrogen atoms [Cd1-N1 = 2.309(7) Å and Cd1-N4 = 2.307(7) Å] from 4AP ligands, one nitrogen atom and one sulfur atom [Cd1-N3 = 2.360(7) Å and Cd1-S1^ii^ = 2.762(2) Å] from isothiocyanate molecules, and one chlorine atom [Cd1-Cl1 = 2.6553(19) Å], thus showing a octahedral coordination geometry [(ii) 1 – x, 1 – y, 1 – z]. The Cd(II) ions are bridged by isothiocyanate molecules and chlorine atoms to generate [Cd_2_(NCS)_2_] and [Cd_2_(Cl)_2_] metalloligands, with Cd⋯Cd separations of 3.99 Å and 5.76 Å. The combination of these metalloligands produces a 1D coordination polymer running parallel to the [100] direction (Figure 4).

Adjacent these 1D coordination polymers are further joined by N-H⋯N, N-H⋯S, and N-H⋯Cl hydrogen bonds (Table S3). The combination of these interactions creates a 3D network in the crystal structure of compound **3** (Figure S1). Compound **3** also contains one C-H⋯π interaction. The C10 atom in the molecule at (x, y, z) acts as a hydrogen-bond donor to the C1-C5/N1 pyridine ring in the molecule at (1/2 – x, 1/2 + y, 1/2 – z), thus forming a C(7) chain running parallel to the [010] direction.

### 3.2. Computational studies of compounds 1–3

The compounds were modeled in Gaussian 03^1^ using the DFT/B3LYP [28] method and LanL2DZ [29] basis set with the SCF = QC convergence approach [30]. The calculations were performed in the gas phase and a vacuum. Coordinates obtained from SC XRD were used as the starting geometry in the theoretical calculations. The structural parameters and some electronic properties of these compounds were obtained. In the theoretical calculations performed for compounds containing transition metals in their structure, the LanL2DZ basis set was used because it gave results in a shorter time and closer to the experimental values than other basis sets. The calculated data of all compounds were visualized using the program GaussView 4.1^2^

#### 3.2.1. HOMO–LUMO energy levels of compounds 1–3

Some of these molecular orbitals are completely filled, some are half filled, and some are completely empty. The highest level of these occupied molecular orbitals is called the HOMO orbital and the lowest level of the empty ones is called the LUMO orbital. The HOMO and LUMO energies of a compound determine the characteristic properties of that compound. The difference between these energy levels also determines the stability of that compound and what color its solutions will be.

**Figure 5:**
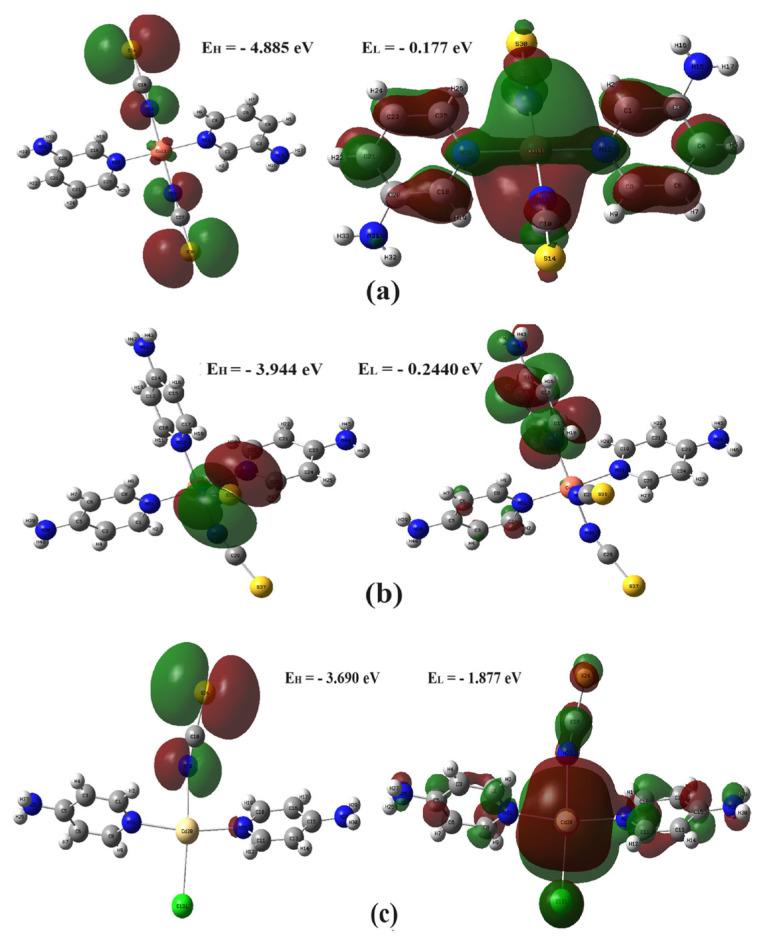
The HOMO and LUMO energy graphs of compounds **1** (a), **2** (b), and **3** (c).

The HOMO and LUMO distributions of all the compounds calculated theoretically by Gaussian 03 are given in Figure 5, from which it can be seen that in compound **1** the HOMO state is located on both Ni(NCS)_2_ groups, in compound **2** only on one Ni(NCS)_2_ group and around the ring nitrogen of the 4AP ligand, and in compound **3** only on its Ni(NCS)_2_ group. In contrast, the LUMO state of compound **1** is distributed almost throughout the compound except for the NH_2_ groups of the 4AP ligand molecule. The LUMO state of compound **2** is dispersed over only one 4AP ligand molecule, which is perpendicular to the other two 4AP ligand molecules in the same plane. The LUMO state in compound **3** is dispersed over nearly the entire compound.

It was determined from the theoretical molecular orbital calculations for the compounds that they have respectively 123, 164, and 109 total orbitals; 78, 103, and 68 fully filled orbitals; 1 half-filled orbital; and 43, 60, and 39 completely empty orbitals.

The formulae used to calculate the electrochemical properties of a compound depending on its HOMO and LUMO values are given as Eqs. (1)–(5).


(1) ΔE=A-I         (Energy gap value)

(2) χ=(I+A)/2=-μ         (Electronegativity, Negative chemical potential)

(3) η=(I-A)/2         (Chemical hardness)

(4) S=1/2η         (Chemical softness)

(5) $$\omega =\mu^{2}/2\eta \;\;\;(\text{Electrophilicity index})$$

The values of the thermochemical properties calculated from the HOMO and LUMO values of the compounds according to Eqs. (1)–(5) are shown in Table 2.

According to Table 2, the following results are available for the chemical activities of the compounds.

➢ The order of the HOMO state energies of the compounds from the largest to the smallest is E_3H_ > E_2H_ > E_1H_. According to this order of values, compound **1** has the strongest electron-donating feature.
➢ The order of the LUMO state energies of the compounds, from largest to smallest, is E_1L_ > E_3L_ > E_2L_. According to this order of values, compound **2** takes the most electrons.
➢ The order of the energy gap value between the HOMO and LUMO states of the compounds, from largest to smallest, is ΔE_1_ > ΔE_2_ > ΔE_3_. In this order, compound **3** has lower kinetic stability, higher chemical activity, and lower semiconductor properties than the other compounds.
➢ The order of electronegativity values of the compounds from largest to smallest is *χ*_3_ > *χ*_1_ > *χ*_2_.
➢ The order of chemical hardness values of the compounds from largest to smallest is *η*_1_ > *η*_2_ > *η*_3_.
➢ The order of chemical softness values of the compounds from largest to smallest is *S*_3_ > *S*_2_ > *S*_1_.
➢ The order of electrophilicity index values of the compounds from largest to smallest is *ω*_3_ > *ω*_2_ > *ω*_1_.

Since the S value of **3** is greater than that of the other compounds and the η value is lower, its intramolecular charge transfer can be expected to be more likely than that of the other compounds.

Some thermochemical properties of the compounds calculated using Gaussian 03 are given in Table 3. These thermochemical properties give very important information about the interactions of these compounds with other compounds.

#### 3.2.2. Other chemical properties of compounds 1–3

The electric dipole moment (μ) of a compound composed of different atoms and molecules is due to the molecular charge distribution of that compound. The μ of a compound has a 3D vector character in 3D space. Therefore, the μ can be used to show the motion of electric charges in a compound. The orientation of the *μ⃗* in a compound in 3D space depends on the location of the centers of the (+) and (−) charges in that compound. The μ of a compound in electrostatic equilibrium is constant and its positioning is precisely determined.

If an external electric field is applied to the electron cloud of any compound in electrostatic equilibrium, its electric charges will move, and its charge balance will be disturbed. This degree of distribution is called “polarizability” for that compound. Often, the term “polarizability” (*α*) is used instead of the term “mean polarizability” (*α*_0_).

The μ, *α*_0_, polarizability anisotropies (Δα), and first-order hyperpolarizability (*β*_0_) values of compounds **1**–**3** were calculated using the LanL2DZ basis set in DFT/B3LYP.

The formulae (6)–(12) were used to obtain the specific μ, *α*_0_, Δα, and *β*_0_values of these compounds, respectively. The μ, *α*_0_, Δα and *β*_0_ values of many chemical compounds have been calculated by the formulae (6)–(12) by various researchers [31,32].


(6) $$\mu =\sqrt{\mu_{x}^{2}+\mu_{y}^{2}+\mu_{z}^{2}}\;\;\;(\text{Dipole moment})$$

(7) $$\alpha_{0}=\frac{\alpha_{xx}+\alpha_{yy}+\alpha_{zz}}{3}\;\;\;(\text{Mean polarizability})$$

(8) $$\Delta \alpha =\sqrt{\frac{{\left(\alpha_{xx}-\alpha_{yy}\right)}^{2}+{\left(\alpha_{yy}-\alpha_{zz}\right)}^{2}+{\left(\alpha_{zz}-\alpha_{xx}\right)}^{2}+6(\alpha_{xy}^{2}+\alpha_{xz}^{2}+\alpha_{zy}^{2})}{2}\;}(\text{Anisotropies of polarizability})$$

(9) $$\beta_{0}=\sqrt{\beta_{x}^{2}+\beta_{y}^{2}+\beta_{z}^{2}}\;\;\;(\text{First-order hyperpolarizability})$$

(10) $$\beta_{x}=\beta_{xxx}+\beta_{xyy}+\beta_{xzz}\;\;\;(\text{x component of})$$

(11) $$\beta_{y}=\beta_{yyy}+\beta_{xxy}+\beta_{yzz}\;\;\;(\text{y component of})$$

(12) $$\beta_{z}=\beta_{zzz}+\beta_{xxz}+\beta_{yyz}\;\;\;(\text{z component of})$$

The calculated μ, *α*_0_, Δα, and *β*_0_ values of the compounds using these formulae are given in Table 4. The Δα, *α*_0_, and *β*_0_ values calculated for the compounds were converted from atomic units (a.u.) to electrostatic units (esu) using the appropriate multipliers [21,32,33].

The order of the calculated values of some magnetic and electrical properties of the compounds from the smallest to the largest is given in Table 4.

➢ *μ*_1_ < *μ*_3_ < *μ*_2_
➢ (Δ*α*)_3_ < (Δ*α*)_1_ < (Δ*α*)_2_
➢ (*α*_0_)_2_ < (*α*_0_)_1_ < (*α*_0_)_3_
➢ (*β*_0_)_1_ < (*β*_0_)_2_ < (*β*_0_)_3_

As a result of these comparisons, it is seen that compound **2** has the highest μ and Δα values and compound **3** the highest *α*_0_ and *β*_0_ values. When the first order hyperpolarization values of the compounds are compared with each other, it is seen that the values of compound **3** and compound **2** are 44.96 and 89.92 times higher than the value of compound **1**, respectively.

#### 3.2.3. Hirshfeld surface analysis of compounds 1–3

In the formation of crystal structures, interactions of shorter reach and less intensity, such as hydrogen–hydrogen and van der Waals interactions, are of great importance, as well as various types of bonds between metallic bonds and other atoms. CrystalExplorer was developed by Spackman et al. to calculate such interactions by using cif files of crystalline compounds [34]. This program can calculate various surface types and properties of a molecular structure such as Hirshfeld, promolecule, crystal voids, electron density, deformation density, electrostatic potentials, molecular orbitals, and spin density [34]. The calculated Hirshfeld surfaces are named in different ways according to their properties [34]. According to the properties of all Hirshfeld surfaces created by CrystalExplorer, they have names such as none, d_i_, d_e_, d_norm_, shape index, curvedness, and fragment patch [34]. The symbols d_i_ and d_e_ in a crystalline molecular structure are the inner and outer distances of the atoms closest to the Hirshfeld surface, respectively. A 3D Hirshfeld surface corresponding to these d_i_ and d_e_ values of a crystal structure is created by CrystalExplorer. A 2D map of a 3D Hirshfeld surface, called a “fingerprint”, is also created by CrystalExplorer. Very important information is obtained from the fingerprint map about the interactions between the nearest neighbor atoms of the molecular structure [34]. While CrystalExplorer calculates and visualizes the voids, curvature radii, and surface values of the voids in almost all crystalline molecular structures, it cannot calculate the interaction energies between the molecules of polymeric crystalline molecular structures [34].

Hirshfeld surface analysis of a molecular structure is a method that determines intermolecular interactions with red, blue, and white regions on that surface. The intensity of the colors on the Hirshfeld surface is directly proportional to the magnitude of the interactions. The red regions show the hydrogen bond interactions, the blue regions show the interactions that are effective up to longer distances, and the white regions show the van der Waals interactions [34].

**Figure 6:**
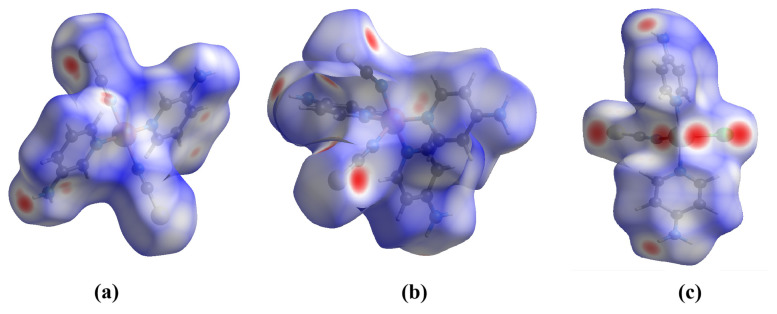
Views of the 3D Hirshfeld surfaces of the unit cells of the compounds **1**: a), **2**: b), and **3**: c) along their different axes.

Intermolecular interactions in the crystal structures of compounds can be determined by Hirshfeld surface analysis. The Hirshfeld surface analysis is a graphic-based tool that facilitates understanding of the interactions between the molecules that make up that molecule in a molecular structure [34]. The views of the Hirshfeld surface created with CrystalExplorer for all three compounds from different angles, the 2D fingerprint graphics produced from them, and the percentage values of various interactions according to all interactions are shown in Figure 6, Figure S2, and Figure S3, respectively.

The Hirshfeld surfaces of the compounds are plotted on their d_norm_ structure in terms of atomic units (a.u.) (Figure 6). The intervals of these Hirshfeld surfaces are from −0.2051 to 1.0140 (**1**), from −0.3470 to 1.3824 (**2**), and from −0.3613 to 1.2572 (**3**) for the compounds (Figure 6).

The 2D projections of the 3D Hirshfeld surfaces seen in Figure 6 describe the interactions that contribute to the formation of the crystal structures of the compounds (Figure S1). These 2D interaction graphs are also known as “fingerprint graphs” of compounds. An examination of Figure S1 reveals that the interactions that have the biggest role in the formation of compounds are C⋯H/H⋯C, S⋯H/H⋯S, H⋯H, and N⋯H/H⋯N interactions. Although the intensities of other interactions are smaller, they are also very important in the formation of the crystal structures of compounds.

Especially in compound **3**, H⋯Cl/Cl⋯H interactions and interactions of many other atoms with the Cl atom and Cl⋯Cl interactions also emerged to a large extent to form the polymeric structure (Figure S2).

**Figure 7:**
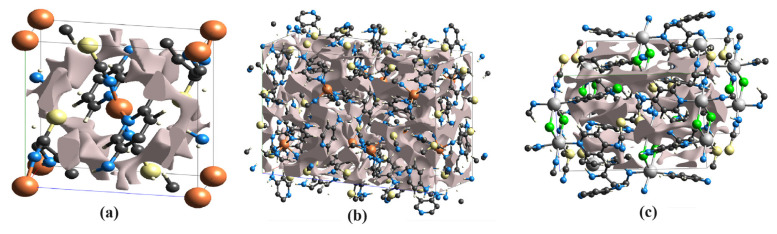
3D views of the voids in the structure of compounds **1** (a), **2** (b), and **3** (c).

Figure 7 shows the voids in the 1 × 1 × 1 sized unit cells of compounds **1**, **2**, and **3**. These void values were obtained from the sum of global atomic electron densities at appropriate nuclear positions in each compound.

To compare the void volume values of the compounds with each other, they are given in Table 5. Examining Table 5 shows that the largest void value is in compound **2**, followed by compound **3** and then compound **1**. The ratios of the void values in the compounds to the volume of the unit cell of the compound also emerged in the same order. According to these values, there are no large voids in any compound.

The durability value of crystal packaging determines its response to mechanical force applied to it externally. If there is a large amount of empty space in a crystal structure, the molecules in that crystal structure are not tightly packed. In this case, that crystal structure can be easily broken by a small external mechanical force. Therefore, the mechanical stability of a crystal structure to be used especially for gas storage must be large enough.

CrystalExplorer 17 can calculate the electrostatic (E_ele_), polarization (E_pol_), dispersion (E_dis_), and exchange-repulsion (E_rep_) energy components in a crystal structure using the CE-B3LYP/6-31G(d,p) and CE-HF/3-21G energy models. Additionally, this program finds the total energy (E_tot_) of that crystal structure by taking the sum of the calculated energy types [34].

The energy components of compounds **1** and **2**, namely electrostatic energies, dispersion energies, others energies, and total energies, were calculated using the CE-HF/3-21G energy model. Since the structure of compound **3** is polymeric, its energy values could not be calculated by CrystalExplorer.

CrystalExplorer determines the values of interaction pairs using crystallographic symmetry. Molecular groups that are identical in terms of symmetry in the crystalline structure are marked with the same colors. This process allows the user of the program to more easily understand the molecular structure and the various interactions occurring in it.

**Figure 8:**
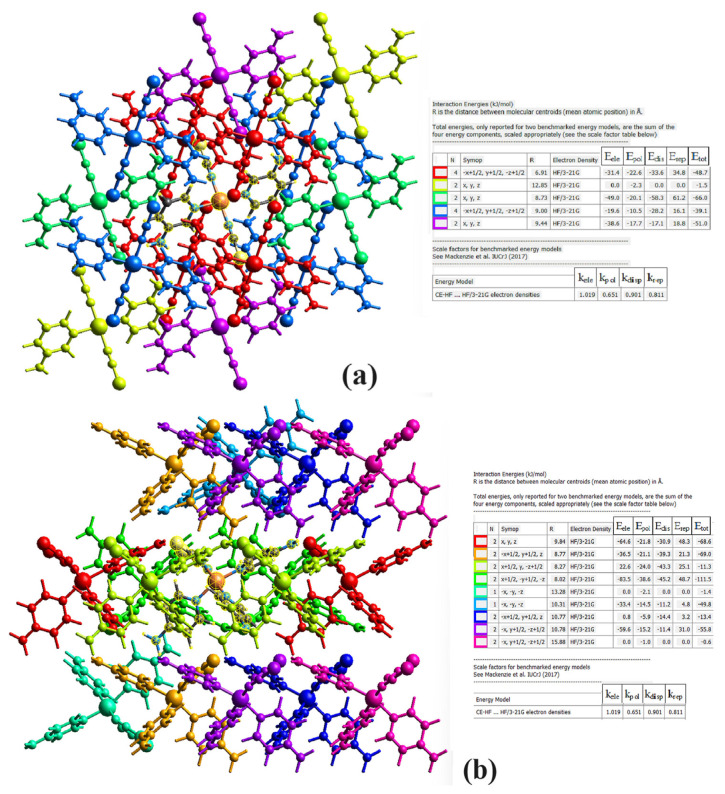
Interaction energies of compound **1** (a) and compound **2** (b) calculated with the HF/3-21G model. In the table of interaction energies, the central molecules are highlighted with a yellow mesh.

The results obtained are given in Figure 8. The energy components of the compounds could not be calculated with this model due to a lack of data in the CE-B3LYP/6-31G(d,p) energy model.

While calculating the total energy values of the compounds with the CE-HF/3-21G energy model, the component energy values were multiplied by the special scale factors seen in Figure 8. In Figures 8a and 8b, it can be seen that the central molecule of compound **1** interacts with 14 neighboring molecules, and the central molecule of compound **2** interacts with 16 neighboring molecules as well.

Examining Figure 8, it can be seen that the interaction with the most dominant total energy value in the formation of compound **1** is the interaction between the green group, which has x, y, and z symmetry operators, and the center molecule, at a value of −66.0 kJ/mol. Similarly, it is seen that the interaction with the most dominant total energy value in the formation of compound **2** is the interaction between the green group, which has x + 1/2, −y + 1/2, and −z symmetry operators, and the center molecule, at a value of −111.5 kJ/mol.

#### 3.2.4. Molecular electrostatic potential (MEP)

To investigate the reactive sites of the compounds, the molecular electrostatic potentials were evaluated using the B3LYP/LanL2DZ method. Figure S4 shows the electrostatic potential map of the molecules. While the points marked in red on this map represent the negative region of electrostatic potential energy, the blue and white parts are regions with low electron density and partial positive charges. The negative (red and yellow) regions of MEP were related to the electrophilic reactivity and the positive (blue) regions to nucleophilic reactivity. As can be seen from Figure S4, the negative regions found around the S and Cl atoms have most of the electron density, especially since their electronegativity is high.

### 3.3. Spectral characterization of compounds 1–3

For comparison with the experimental vibration frequencies of the compounds, their theoretical vibration frequencies were calculated theoretically with the DFT/B3LYP method and LanL2DZ basis set in the gas phase [28]. The spectra obtained are given together in Figure S5.

When the spectra of the compounds are compared with each other, some similarities are seen between them. These similarities arise from the presence of the same or very similar ligands in the structures of the compounds. Some differences between the spectra of the compounds arise from the presence of different transition metal atoms in their structures and the positioning of various ligand molecules in the structure of the compounds.

All experimental data of the compounds confirm the presence of 3AP, 4AP, (NCS)^−^, and Cl^−^ ligands and transition metal atoms such as Cu and Cd in their structures. Spectral graphs of the compounds were analyzed in detail for the vibrations of 3AP, 4AP, and (NCS)^−^ ligands.

#### 3.3.1. Vibrations of 3AP and 4AP ligands in compounds 1–3

Many researchers have published studies on 3AP and 4AP ligand molecules, their structural properties, and the compounds they form with various metals [9–12,14,15,17–21,35–37]. In the aforementioned studies, new compounds were obtained, mostly in powder form and a few in crystals form, by using 3AP and 4AP ligands. In the present study, three crystalline compounds obtained using 3AP, 4AP, (NCS)^−^, and Cl^−^ ligand molecules and Cu(II) and Cd(II) transition metal atoms were examined in terms of their various properties.

The vibration modes of the 3AP and 4AP ligand molecules in compounds **1**–**3**, and the changes in these modes resulting from the formation of the crystal structure are given in Table 6.

##### 3.3.1.1. Vibrations of 3AP ligand in compound 1

Table 6 gives the vibrations of the 3AP ligand molecule in the free state and in compound **1**. These vibration values are consistent with those in our and other researchers’ previous studies [14,19,35–37].

Some important vibration modes of the 3AP ligand molecule in compound **1**, which are affected by the formation of the crystal structure of the compound, were observed at wavenumbers ν_as_(NH_2_) = 3459, ν_s_(NH_2_) = 3350, δ(NH_2_) = 1609, ν_ring_ = 1275, γ_ring_ + γ(CH) = 695, and δ_ring_ = 655 cm^−1^ [33–35]. The shift amounts of these vibration modes to high wavenumber (+) or low wavenumber (−) compared to its free state values are +86, +46, −24, +15, −7, and +32 cm^−1^, respectively. Shifts in these vibration modes compared to the free state have also been observed in previous studies [35–37].

##### 3.3.1.2. Vibrations of 4AP ligand in compounds 2 and 3

Additionally, Table 6 gives the vibrations of the 4AP ligand molecule in the free state and in compounds **2** and **3**.

Some important vibration modes of the 4AP ligand molecule in compound **2**, which are affected by the formation of the crystal structure of the compound, were observed at wavenumbers ν_as_(NH_2_) = 3472, ν_s_(NH_2_) = 3404, ν_s_(C-H) = 3114, ν_as_(C-H) = 3069, δ(NH_2_) = 1633, ν(C=N) = 1559, ν_ring_ = 1516, ν(C-C) = 1453, ν(C-NH_2_), ν(C-C) = 1344, ν(C-N) = 1283, γ(C-C-C), ring breath. = 1021, and β(C-H) = 823 cm^−1^.

The shifts of these vibration modes of the 4AP ligand molecule in compound **2** to high wavenumber (+) or low wavenumber (−) compared to its free state values are +42, +101, +14, −10, −16, +35, +22, +24, +15, +16, +45, and +10 cm^−1^, respectively. Shifts in these vibration modes compared to the free state have also been observed in previous studies [17–21].

Similarly, some important vibration modes of the 4AP ligand molecule in compound **3**, which are affected by the formation of the crystal structure of the compound, were observed at wavenumbers ν_as_(NH_2_) = 3448, ν_s_(NH_2_) = 3349, ν_s_(C-H) = 3119, ν_as_(C-H) = 3068, δ(NH_2_) = 1624, ν(C=N) = 1562, ν_ring_ = 1515, ν(C-C) = 1449, ν(C-NH_2_), ν(C-C) = 1353, ν(C-N) = 1280, γ(C-C-C), ring breath. = 1013, and β(C-H) = 824 cm^−1^.

The amount of shift of some vibration modes of the 4AP ligand molecule to high wavenumber (+) or low wavenumber (−) in compound **3**, as in compound **2**, according to its free state values are +18, +46, +19, −11, −25, +38, +21, +20, +24, +12, +37, and +11 cm^−1^, respectively. Changes in these vibration modes compared to the free state have also been observed in previous studies [17–21].

When Figure S5 and Table 6 are examined carefully, it can be seen that there is a discrepancy in the results. The reason for this is that while the experimental FTIR spectra of compounds are obtained from an almost infinite number of unit structures interacting with each other in the same structure, the theoretical FTIR spectrum is obtained from a unit structure alone and in the gas phase. Therefore, the theoretical spectrum is simpler than the experimental spectrum and some vibration frequencies occur in very different places. If the number of unit structures in the theoretical calculation is increased, the appearances of the theoretical and experimental spectra become closer and closer to each other.

##### 3.3.1.3. IR active vibrations of (NCS) ligand in compounds 1–3

Many scientists have conducted various studies on the compound KNCS [39–45]. According to theoretical and experimental studies, the (NCS)^−^ ion has three vibration modes. Two of these vibration modes are stretching and one is bending vibration mode, and they are expressed with the symbols ν(NC), ν(CS), and δ(NCS), respectively.

To see the effects of compound formation on the characteristic vibration modes of the (NCS)^−^ ion group, the characteristic vibration modes of the compounds and KNCS are given comparatively in Table 7. The assignment of characteristic vibration modes of KNCS in Table 7 is based on Jones’ work on this subject [38].

While the characteristic ν(NC) mode in KNCS was observed at a wavenumber of 2036 cm^−1^, this mode was affected by the formation of compounds **1** and **2** and shifted to higher wavenumbers of 50 and 44 cm^−1^, respectively (see Table 7). In compound **3**, the same vibration mode was affected by its formation and shifted to a higher wavenumber of 68–34 cm^−1^ and split in two (see Table 7). This vibration mode is in the triple bond category according to its wavenumber value. The bonding character of the CN bond is also confirmed by its bond lengths in compounds. From the crystal data of the compounds, the NC bond distances obtained in the isothiocyanate structure were very close to the average value of 1.135 Å and the N-M (M = Cu, Cu, and Cd) distances obtained were very close to the average value of 2.114 Å. This result shows that the character of the ν(NC) vibration mode in the isothiocyanate structures is very close to the triple bond. According to our study, the dependence of these shifts towards higher wavenumbers on transition metal atoms was Cd > Cu.

Another characteristic vibration mode of KNCS is observed at wavenumber ν(CS) 740 cm^−1^. This vibration mode was affected by compound formation as well. This mode shifted to higher wavenumbers of 166–142 cm^−1^ in the new compounds obtained (see Table 7). The dependence of these high wavenumber shifts in the compounds on the transition metal atoms was Cd > Cu.

Another characteristic vibration mode of the free KNCS is the δ(NCS) bending mode. This vibration mode appears in the IR spectrum of KNCS as two degenerate modes at wavenumbers of 474 and 484 cm^−1^. However, this vibration mode was also affected by compound formation and emerged as a single mode in the compounds at wavenumbers of 469, 489, and 478 cm^−1^, respectively (see Table 7).

The 2δ(NCS) vibration mode, which is the overtone of the δ(NCS) characteristic bending vibration mode of the free KNCS, was observed as a single weak peak at a wavenumber of 957 cm^−1^ in its IR spectrum. This mode was observed as a single weak peak in compounds **1**, **2**, and **3** at wavenumbers of 934, 942, and 935 cm^−1^, respectively.

##### 3.3.1.4. Theoretical UV and visible spectra of compounds

Theoretical UV–vis absorption spectra of the compounds were analyzed (for N_state_ = 30) using the TD-SCF, B3LYP/LanL2DZ [scrf = (iefpcm, solvent = water)] theoretical basis set. For each compound, a number of electronic transitions were calculated at 30 excitation levels. The UV–vis data of the compounds were obtained by drawing interconnected graphs of the wavelengths (λ, in nm) and oscillation strengths (f, unitless) of each of these 30 excitation levels (see Figures S6a–S6c) [31]. These electronic transitions of the compounds are given in Table S4 along with various properties of them.

According to Figure S6 and Table S4, compounds **1**, **2**, and **3** most significantly have 5 UV and 1 vis transitions, 4 UV and 1 vis transitions, and 9 UV transitions, respectively. Some of these transitions may not be observed in the experimental spectrum because either they require too much energy or their intensities are too small. In fact, due to the type and pH value of the solvent environment, these peaks broaden and overlap each other. As a result, the UV spectrum of a compound can be obtained as a very broad single peak [45].

Transitions between approximately 210 and 290 nm in each compound generally correspond to π → π* and n → π* transition types, while transitions between 300 and 360 nm can be considered to correspond to ligand-to-metal (L→M) charge transitions [46,47]. Generally, transitions between 400 and 800 nm are attributed to the interaction of radiation in the visible region with the compound [45].

**Figure 9:**
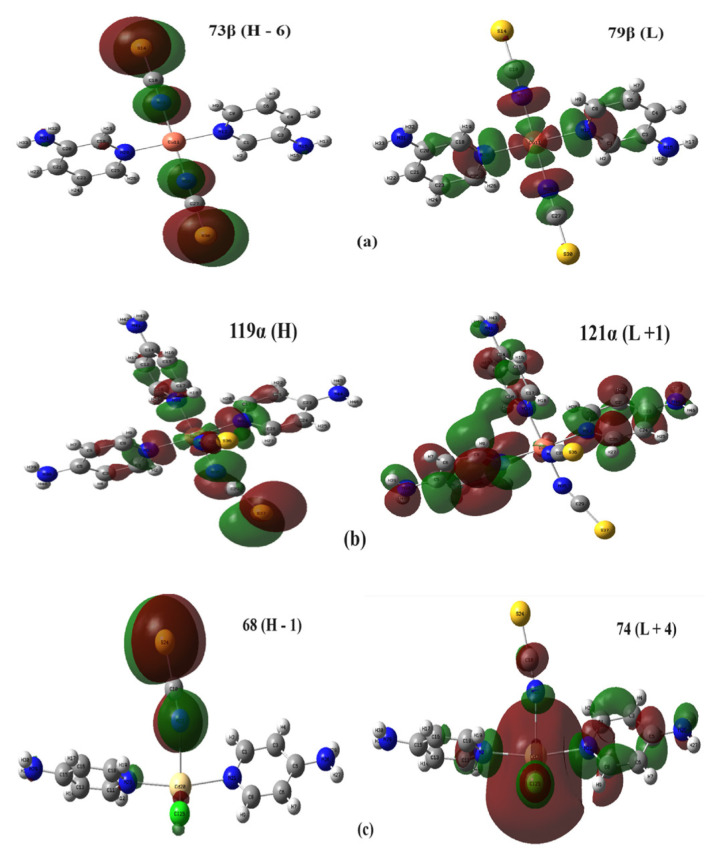
Examples of UV excitation transitions of compounds **1** (a), **2** (b), and **3** (c).

In order to serve as an example of the UV excitation transitions of compounds **1**–**3** in Table S4, the charge distribution forms of those with the largest C_i_ coefficient in each compound are given in Figure 9. These transitions for the compounds are, respectively, 73β → 79β, 119α →121α, and 68 → 74.

Examining Figure 9, it can be seen that in compounds **1** and **3** there is generally a charge transition from the isothiocyanate ligands to the Cu and Cd atoms, respectively, and in compound **2** there is generally a charge transition between the ligands.

### 3.4. Thermal behavior of compounds 1–3

The thermal behavior of the compounds was investigated in the temperature range of 30 to 950 °C. Thermal behavior graphs for each compound are shown in Figures S7a–S7c, respectively. From the TGA, DTG, and DTA thermal behavior curves of the compounds, it is seen that their crystal structures remain intact from room temperature to approximately 200 °C (see Figure S7).

A brief summary of the thermal behavior of the compounds is listed in Table S5 for easier comparison. An examination of Table S5 reveals that all compounds undergo thermal decomposition in three main steps in the temperature range of approximately 200 to 900 °C. In the first step of this thermal degradation, the compounds gradually lose the 3AP and 4AP ligand molecules in their crystal structures. In the second step, the compounds lose the isothiocyanate and chlorine ligand molecules in their structures. In the last thermal step, only the Cu and Cd transition metal atoms in the compounds remain in the environment. The experimental thermal degradation results of the compounds are in very good agreement with their theoretically obtained thermal degradation results. The thermal degradation steps of the compounds are in accordance with those in previous similar studies [47–52].

The following information can be given about the thermal behavior of the compounds from Figure S7 and Table S5, respectively.

For compound **1**: The crystal structure of compound **1** is preserved intact in the temperature range of 30–170 °C. As the ambient temperature increases, compound **1** releases two 3AP ligand molecules in its structure into the external environment in two stages within the temperature range of 170–395 °C (DTG_max_ = 245 °C and DTG_max_ = 326 °C). As the temperature increases further, compound **1** releases two NCS ligand molecules in its structure into the external environment in two stages within the temperature range of 395–925 °C (DTG_max_ = 501 °C and DTG_max_ = 680 °C). At temperatures higher than 925 °C, only Cu transition metal atoms from compound **1** remain in the environment.

For compound **2**: The crystal structure of compound **2** is preserved intact in the temperature range of 30–205 °C. As the ambient temperature increases, compound **2** releases three 4AP ligand molecules in its structure into the external environment in three stages within the temperature range of 205–347 °C (DTG_max_ = 278 °C, DTG_max_ = 306 °C, and DTG_max_ = 334 °C). As the temperature increases further, compound **2** releases two NCS ligand molecules in its structure into the external environment in two stages within the temperature range of 347–915 °C (DTG_max_ = 420 °C and DTG_max_ = 672 °C). At temperatures higher than 915 °C, only Cu transition metal atoms from compound **2** remain in the environment.

For compound **3**: The crystal structure of compound **3** is preserved intact in the temperature range of 30–190 °C. As the ambient temperature increases, compound **3** releases the other parts of the two 4AP ligand molecules in its structure, except the ring nitrogen atoms, into the external environment in two stages within the temperature range of 190–385 °C (DTG_max_ = 217 °C and DTG_max_ = 254 °C). As the temperature increases further, compound **3** releases one NCS ligand molecule, one Cl atom, and the remaining two N atoms from 4AP molecules into the external environment in four stages within the temperature range of 385–750 °C (DTG_max_ = 441 °C, DTG_max_ = 458 °C, DTG_max_ = 510 °C, and DTG_max_ = 587 °C). At temperatures higher than 750 °C, only Cd transition metal atoms from compound **3** remain in the environment.

In examining the thermal processes of all three compounds, the realization values of the decomposition steps are in close agreement both theoretically and experimentally (see Table S5). As a result of the thermal analysis of the compounds, it was seen that their thermal decomposition steps supported their crystal structures.

## 4. Conclusion

In the present study, three complexes were synthesized in crystal form for the first time. The compounds were characterized by various spectroscopic methods such as FTIR spectroscopy, elemental, and SC-XRD analysis. According to the SC-XRD results, the ligand molecules in the complexes made single bonds from the ring nitrogen to the metal atoms. The compounds showed that the first of these coordination compounds had a 1D crystal structure and the other two had a 3D crystal structure. N-H⋯S, N-H⋯N, N-H⋯Cl, N-H⋯π, and C-H⋯π bonds and their combinations were effective in the formation of the crystal structures of the said coordination compounds. The metal atoms [Cu(II), Cu(II), and Cd(II)] in these coordination compounds were surrounded by various ligand molecules in a square planar, square pyramidal, and octahedral arrangement, respectively. Theoretical information about the compounds was obtained using Gaussian 03 and CrystalExplorer. The theoretical results obtained for the compounds were compatible with the experimental results. When the energy gap value between the HOMO and LUMO states of the compounds was examined, it was predicted that compound **3** may have lower kinetic stability, higher chemical activity, and lower semiconductor properties than all other compounds. Since the crystal structures of the compounds are transparent and permeable to light, it is recommended that relevant experts examine their optoelectronic properties in detail. In addition, since the crystal void value of compound **2** is the largest, it is recommended to investigate its ability to store various gases.

## Supplementary Data

Figure S1 An infinite 3D layer in **3**.

Figure S2 2D fingerprint graphs obtained from the projections of some of the highest value intermolecular interactions of compounds **1**, **2**, and **3** on the d_norm_ Hirshfeld surface.

Figure S3 Percentage contributions of various intermolecular interactions of compounds **1**, **2**, and **3** in 2D fingerprint plots ordered from largest to smallest.

Figure S4 Molecular electrostatic potential maps of I, II, and III molecules calculated at DFT/B3LYP/LanL2DZ level.

Figure S5 Experimental and theoretical FTIR spectra of compounds **1** (a), **2** (b), and **3** (c).

Figure S6 Theoretical UV–vis graphs of compounds **1** (a), **2** (b), and **3** (c).

Figure S7 TGA and DTG curves of the thermal behavior of compounds **1** (a), **2** (b), and **3** (c).

**Table S1:** Elemental analysis results of the compounds.

Compounds	Elemental analysis, (calculated) (%)/found (%)					
	Metal	N	C	S	H	M _r_ (g)
[Cu(C _5_H _6_N _2_) _2_(NCS) _2_]	(17.27) 16.94	(22.84) 21.91	(39.17) 39.72	(17.43) 16.71	(3.29) 4.03	367.94
[Cu(C _5_H _6_N _2_) _3_(NCS) _2_]	(13.75) 13.17	(24.25) 24.51	(44.19) 43.26	(13.88) 12.97	(3.93) 4.29	462.05
[Cd(C _5_H _6_N _2_) _2_(NCS)Cl]	(28.52) 27.94	(17.77) 17.06	(33.52) 32.49	(8.13) 7.67	(3.07) 3.43	394.13

**Table S2:** Crystal data and structure refinement parameters for compounds **1**–**3**.

Crystal data	1	2	3
Empirical formula	C _12_H _12_CuN _6_S _2_	C _17_H _18_CuN _8_S _2_	C _11_H _12_CdClN _5_S
Formula weight	367.94	462.05	394.17
Crystal system	Monoclinic	Orthorhombic	Monoclinic
Space group	P2 _1_/n	Pbca	P2 _1_/n
*a (*Å)	8.7272 (8)	9.8440 (11)	9.3538 (9)
*b (*Å)	9.4363 (10)	16.525 (2)	16.2760 (16)
*c (*Å)	9.6050 (11)	25.929 (3)	9.7326 (10)
*b (*°)	113.363 (5)	90.00	105.605 (4)
*V (*Å ^3^)	726.14 (13)	4218.1 (9)	1427.1 (2)
Z	2	8	4
*D* _c_ (g cm ^−3^)	1.683	1.455	1.835
θ range (°)	3.2–28.3	2.9–26.4	3.0–26.2
Measured reflections	19520	62689	54873
Independent reflections	1331	4105	3550
*R* _int_	0.060	0.10	0.05
S	1.04	1.14	1.01
R1/wR2	0.076/0.183	0.094/0.174	0.072/0.206
Dr _max_/Dr _min_ (eÅ ^−3^)	0.49/−0.71	0.45/−0.51	0.72/−0.87
CCDC	2245812	2245813	2245814

**Table S3:** Hydrogen-bond parameters for **1**–**3** (Å, °).

D-H· · ·A	D-H	H⋯A	D⋯A	D-H⋯A
**Compound 1**				

N2—H2B⋯S1 ^ii^	0.86 (2)	2.75 (4)	3.587 (9)	165

**Compound 2**				

C10—H10⋯N7	0.93	2.58	3.154 (11)	120
N2—H2A⋯S1 ^i^	0.86 (2)	2.66 (3)	3.490 (7)	164
N2—H2B⋯S2 ^i^	0.86 (2)	2.71 (3)	3.554 (7)	168
N4—H4A⋯S1 ^ii^	0.86 (2)	2.55 (4)	3.397 (14)	168
N6—H6B⋯S2 ^iii^	0.86 (2)	2.63 (4)	3.473 (9)	165

**Compound 3**				

N2—H2A⋯S1 ^iii^	0.86 (2)	2.82 (5)	3.597 (8)	152
N2—H2B⋯Cl1 ^iv^	0.86 (2)	2.62 (5)	3.432 (7)	158
N5—H5A⋯N3 ^v^	0.86 (2)	2.55 (5)	3.365 (12)	159
N5—H5B⋯Cl1 ^vi^	0.86 (2)	2.66 (3)	3.504 (8)	168

Symmetry codes: (ii) −x+2, −y+1, −z+1 for **1**; (i) x+1, y, z; (ii) −x+1, y−1/2, −z+3/2; (iii) x−1/2, −y+3/2, −z+1 for **2**; (iii) −x+1/2, y−1/2, −z+1/2; (iv) x+1/2, −y+1/2, z+1/2; (v) −x+1/2, y+1/2, −z+1/2; (vi) x+1/2, −y+3/2, z+1/2 for **3**.

**Table S4:** Theoretically calculated absorption wavelength (λ), excitation energies, and oscillator strengths (f) of compounds **1**–**3**.

Complexes	Excitation states	Excitation energy (eV)	Wavelength (nm)	Oscillation strengths	Assignments	Coefficients C _i_

					From → to	
**1**	1	4.4559	278.25	0.0157	76β → 80β	0.67602
	2	4.3922	282.28	0.0785	78α → 80α	0.61554
	3	4.0719	304.48	0.0080	71β → 79β	0.98453
	4	3.7789	328.10	0.4081	78α → 83α	0.48852
	5	2.3456	528.59	0.0099	73β → 79β	0.99661
	6	2.0431	606.83	0.0093	78β → 79β	0.99574

**2**	1	4.7474	261.16	0.0098	117β →119β	0.54398
	2	4.5822	270.57	0.0264	116β →119β	0.42566
	3	4.5024	275.38	0.0064	118β →120β	0.61858
	4	4.4573	278.16	0.0012	119α →121α	0.74355
	5	1.4824	836.39	0.0005	110β →119β	0.63326

**3**	1	5.8944	210.34	0.0038	63 → 70	0.50522
	2	5.8258	212.82	0.0057	66 → 74	0.36574
	3	5.7873	214.23	0.0873	65 → 72	0.39290
	4	5.6706	218.64	0.1655	67 → 70	0.41138
	5	5.6478	219.53	0.1556	66→ 72	0.35218
	6	5.6046	221.22	0.1256	66 → 73	0.36270
	7	5.4831	226.12	0.0143	68 → 74	0.66020
	8	5.2292	237.10	0,0198	67 → 73	0.34919
	9	5.0216	246.90	0.0079	69 → 71	0.58510

**Table S5:** Thermal analyses results of compounds **1**–**3**.

Compound	Temperature range (°C)	DTG _max_ (°C)	Group leaving the compound	Calculated (%)	Found (%)
**1**	170–395	245, 326	2(3AP)	51.16	51.20
	395–925	501, 680	2(SCN)	31.57	31.50
	925–950	-	Cu	17.27	17.30
**2**	205–347	278, 306, 334	3(4AP)	61.11	60.92
	347–915	420, 672	2(SCN)	25.14	25.58
	915–950	-	Cu	13.75	13.50
**3**	190–385	217, 254	C _10_H _12_N _2_	40.65	40.40
	385–750	441, 458, 510, 587	C _1_ClN _3_S	30.83	30.90
	750–950	-	Cd	28.52	28.70

## Figures and Tables

**Table 1:** Selected bond distances and angles for compounds **1**–**3** (Å, °).

**Compound 1**			

Cu1-N1	2.013(6)	Cu1-N3	1.963(7)
N3-Cu1-N1^i^	89.6(3)	N3-Cu1-N1	90.4(3)

**Compound 2**			

Cu1-N1	2.006(6)	Cu1-N3	2.018(5)
Cu1-N5	2.028(5)	Cu1-N7	2.175(7)
Cu1-N8	2.018(6)		
N1-Cu1-N8	91.0(2)	N1-Cu1-N3	88.6(2)
N8-Cu1-N3	171.1(3)	N1-Cu1-N5	160.7(2)
N8-Cu1-N5	87.9(2)	N3-Cu1-N5	89.5(2)
N1-Cu1-N7	100.8(3)	N8-Cu1-N7	93.0(3)
N3-Cu1-N7	95.8(2)	N5-Cu1-N7	98.4(3)

**Compound 3**			

Cd1-N1	2.309(7)	Cd1-N3	2.360(7)
Cd1-N4	2.307(7)	Cd1-Cl1	2.6553(19)
Cd1-S1^ii^	2.762(2)		
N4-Cd1-N1	168.7(2)	N4-Cd1-N3	86.3(3)
N1-Cd1-N3	83.1(3)	N4-Cd1-Cl1	91.68(16)
N1-Cd1-Cl1	93.20(16)	N3-Cd1-Cl1	93.9(2)
N4-Cd1-Cl1^i^	97.17(16)	N1-Cd1-Cl1^i^	93.50(16)
N3-Cd1-Cl1^i^	176.1(2)	Cl1-Cd1-Cl1^i^	84.21(6)
N4-Cd1-S1^ii^	89.82(16)	N1-Cd1-S1^ii^	87.71(16)
N3-Cd1-S1^ii^	99.0(2)	Cl1^i^-Cd1-S1^ii^	82.82(6)

Symmetry codes: (i) −x+1, −y+1, −z+1 for **1**; (i) −x+1, −y+1, −z; (ii) −x+1, −y+1, −z+1 for **3**.

**Table 2:** The HOMO, LUMO, and chemical efficiency values in (eV) units of compounds **1**–**3**.

Chemical efficiency values	1	2	3
E_HOMO_ (−I)	−4.885	−3.944	−3.690
E_LUMO_ (−A)	−0.177	−0.244	−1.877
ΔE = A − I	4.708	3.700	1.813
χ	2.531	2.094	2.784
μ	−2.531	−2.094	−2.784
η	2.354	1.850	0.907
S (eV)^−1^	0.212	0.270	0.552
ω	1.361	1.185	4.274

**Table 3:** Some thermochemical data of compounds **1**–**3**.

		1	2	3
E (kcal/mol)	Electronic	0.000	0.000	0.000
	Translational	0.889	0.889	0.889
	Rotational	0.889	0.889	0.889
	Vibrational	164.274	241.015	165.078
	Total	166.050	242.790	166.860

Heat capacity at constant volume C_v_ (cal/mol-Kelvin)	Electronic	0.000	0.000	0.000
	Translational	2.981	2.981	2.981
	Rotational	2.981	2.981	2.981
	Vibrational	41.028	58.579	58.165
	Total	46.990	64.540	64.130

Entropy S (cal/mol-Kelvin)	Electronic	1.377	1.377	1.377
	Translational	43.594	44.274	45.474
	Rotational	35.464	36.618	37.926
	Vibrational	42.280	62.084	75.456
	Total	122.720	144.350	160.230

Zero-point vibrational energy E_v0_	(Joules/mol)	659795.600	968085.000	645580.700
	(kcal/mol)	157.695	231.378	154.298

Rotational constants (GHz)	A	0.25135	0.14398	0.11711
	B	0.17143	0.11420	0.06872
	C	0.11078	0.09086	0.04980

**Table 4:** The electric dipole moment, anisotropies of polarizability, the mean polarizability, and the first order hyperpolarizability of compounds **1**–**3**.

	1	2	3
μ_x_ (D)	0.0293	−10.1405	1.3769
μ_y_ (D)	−0.0930	17.6945	−7.1328
μ_z_ (D)	0.4393	−11.6188	−9.1954
μ (D)	0.450	23.472	11.719
α_xx_ (a.u.)	−64.9337	−98.9931	−12.8747
α_xy_ (a.u.)	30.1418	32.0890	1.5785
α_yy_ (a.u.)	−215.7345	−172.4887	−178.9911
α_xz_ (a.u.)	−13.2816	−10.4307	−3.0965
α_yz_ (a.u.)	17.7210	6.0641	1.7535
α_zz_ (a.u.)	−127.7191	−209.4666	−131.9920
Δα (esu)	2.169 × 10^−23^	1.691 × 10^−23^	2.200 × 10^−23^
(esu)	−2.017 × 10^−23^	−2.376 × 10^−23^	−1.599 × 10^−23^
β_xxx_ (a.u.)	−3.0791	−100.2083	27.9845
β_xxy_ (a.u.)	1.9699	63.7319	−39.9522
β_xyy_ (a.u.)	2.6175	−191.1888	41.5301
β_yyy_ (a.u.)	−5.4200	416.2235	−225.2250
β_xxz_ (a.u.)	4.8247	−112.6955	−65.3111
β_xyz_ (a.u.)	0.4364	−52.1285	−17.3862
β_yyz_ (a.u.)	1.9458	−9.2955	−53.2764
β_xzz_ (a.u.)	0.1310	−66.0282	−9.2596
β_yzz_ (a.u.)	0.2478	57.3615	−6.8830
β_zzz_ (a.u.)	1.4346	−248.2134	−51.3244
(esu)	6.272 × 10^−32^	5.640 × 10^−30^	2.820 × 10^−30^

**Table 5:** General characteristics of the voids in the unit cells of complexes **1**, **2**, and **3**.

Complexes	Volume (Å^3^)	RVVTV (%)	Void area (Å^2^)	Globularity	Asphericity
**1**	31.49	04.34	164.20	0.294	0.104
**2**	566.88	13.44	1660.22	0.200	0.222
**3**	123.67	08.67	467.88	0.257	0.122

RVVTV (%). Ratio of void volume to total volume (%).

**Table 6:** Experimental and theoretical vibration absorption wavenumbers (cm^−1^) of 3AP and 4AP ligand molecules in the free state and in crystalline complexes **1**–**3**.

Ligand molecules									
3AP				4AP					
Assignment a	F.S.	In **1**		Assignment b	F.S.	In **2**		In **3**	
		Exp	Theor			Exp	Theor	Exp	Theor
ν_as_(NH_2_)	3373 s	3459 s	6221 s	ν_as_(NH_2_)	3430 s	3472 m	6238 w	3448 s	6280 w
ν_s_(NH_2_)	3304 m	3350 s	5836 s	ν_s_(NH_2_)	3303 w	3404 w	6218 w	3349 s	5943 s
ν(C-H)	3066 w	3061 w	5160 s	ν_s_(C-H)	3100 w	3114 w	5881 m	3119 w	5879 m
ν(C-H)	3035 w	3041 w	5133 m	ν_as_(C-H)	3079 w	3069 w	5826 m	3068 w	5165 vw
δ(NH_2_)	1633 m	1609 s	1791 m	ν_as_(C-H)	3036 w	n.o.	5134 vw	n.o.	5125 w
ν_ring_ + δ(CH)	1584 s	1579 s	1574 vw	ν(C-H)	2986 w	2973 w	5116 vw	n.o.	n.o.
ν_ring_	1486 s	1506 w	1508 vw	δ(NH_2_)	1649 m	1633 s	1786 s	1624 s	1776 m
δ(CH) + ν_ring_	1433 s	1449 s	n.o.	ν(C=C), γ(C-C-C)	1585 s	n.o.	1608 vw	n.o.	n.o.
δ(CH)	1347 w	1348 w	1349 vw	ν(C=N)	1524 w	1559 m	n.o.	1562 m	n.o.
ν_(C-NH2)_ + ν_ring_	1291 s	1315 s	n.o.	ν_ring_	1494 m	1516 s	1515 vw	1515 s	1507 vw
ν_ring_	1260 s	1275 s	n.o.	ν(C-C)	1429 w	1453 m	1416 w	1449 m	1400 vw
δ(CH) + ν_ring_	1195 w	1191 m	n.o.	ν(C-NH_2_), ν(C-C)	1329 w	1344 m	1382 vw	1353 m	n.o.
δ(CH)+ν_ring_+δ(CNH)	1125 m	1135 s	n.o.	ν(C-N)	1267 m	1283 m		1280 m	n.o.
NH_2_ twist + ν_ring_	1091 m	1120 m	1122 vw	γ(C-H)	1204 m	1212 s		1215 s	1112 vw
Ring breath, ν_ring_ +δ(CH)	1042 m	1057 m	n.o.	γ(C-H), γ(C-C-C)	1055 w	1056 m		1057 m	n.o.
δ_ring_ + ν_ring_	1014 w	1022 w	1000 vw	γ(C-C-C), ring breath.	976 s	1021 s		1013 s	n.o.
γ(CH)	963 m	n.o.	994 vw	ω(NH_2_), τ(NH_2_)	838 sh	847 sh		851 m	
γ(CH)	908 w	934 w	976 vw	β(C-H)	813 s	823 s		824 s	
γ(CH)	871 m	882 s	n.o.	γ(C-C-C), ring def.	664 s	665 w		668 w	757 vw
γ(CH)+δ_ring_+γ_(C-NH2)_	843 m	n.o.	n.o.	γ(CNC),ω(ring),ρ(NH_2_)	524 s	528 s		529 s	
γ(CH)+γ_ring_+γ_(C-NH2)_	797 s	796 w	782 vw	γ(C-NH_2_)	442 w	445 w		445 w	
γ_ring_ + γ(CH)	702 s	695 s	723 vw						
δ_ring_	623 m	655 s	n.o.						
δ_ring_+NH_2_wag+γ_(CNH2)_	537 w	551 m	572 vw						
NH_2_wag+γ_(C-NH2)_+γ_ring_	510 w	536 w	n.o.						
γ_(C-NH2)_ + NH_2_wag + γ_ring_	453 w	469 m	443 vw						

ν: stretching; δ: out of plane bending; γ: in plane bending; β: out of plane bending; ω: wagging; τ: torsion; ρ: rocking; s: strong; m: medium; w: weak; F.S.: free state; n.o.: not observed, Exp: experimental; Theor: theoretical.

a Taken from Ref. [34],

b Taken from Ref. [21].

**Table 7:** Fundamental FTIR vibration wavenumbers (cm^−1^) of solid KNCS and the isothiocyanate group (NCS)^−^ in compounds **1**–**3**.

Assignment a	ν̄^a^	Compounds		
		1	2	3
ν(NC)	2036 s	2096 s	2084 s	2104 s 2070 s
ν(CS)	740 m	882 s	892 w	906 w
δ(NCS)	484 w 474 s	469 m	489 w	478 w
2δ(NCS)	957 m	934 w	942 w	935 w

ν: stretching; δ: out of plane bending; s: strong; m: medium; w: weak.

a Taken reference [38].

## Footnotes

1 Frisch MJ, Trucks GW, Schlegel HB, Scuseria GE, Robb MA et al. Gaussian 03, Revision D. 01. Gaussian, Inc.; 2004.

2 Dennington R, Keith T, Millam J. Gauss View, Version 4.1.2. Semichem Inc.; 2007.
