# Supplementary figures and images for: Synthesis, characterization, and Hirshfeld surface analysis of coordination compounds composed of two different aminopyridines, isothiocyanate ligand molecules, and two different transition metal atoms
Source: Turk J Chem. 2024 Sep 17;48(5):780–99. doi: 10.55730/1300-0527.3697 (PMC11539913; doi:10.55730/1300-0527.3697)

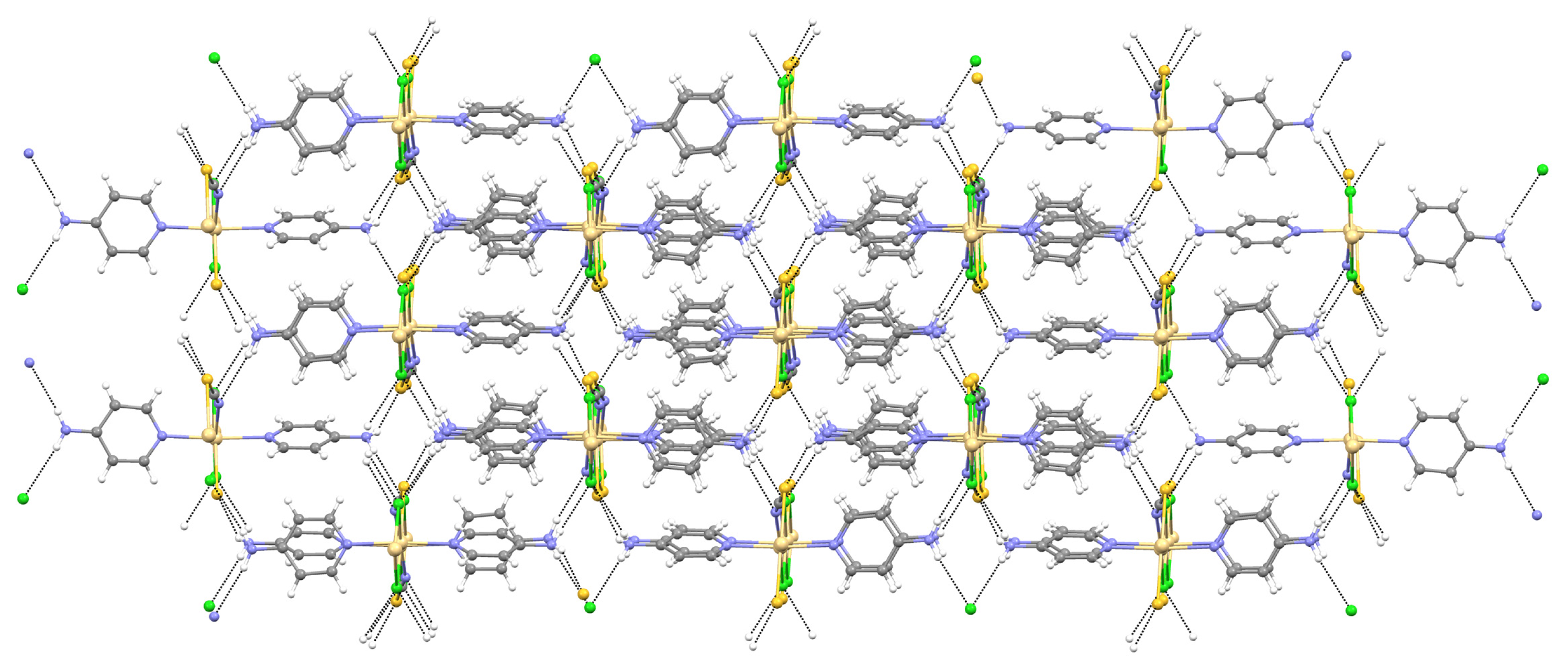

Supplement: Figure S1 — An infinite 3D layer in 3. [file tjc-48-05-780s1.tif]

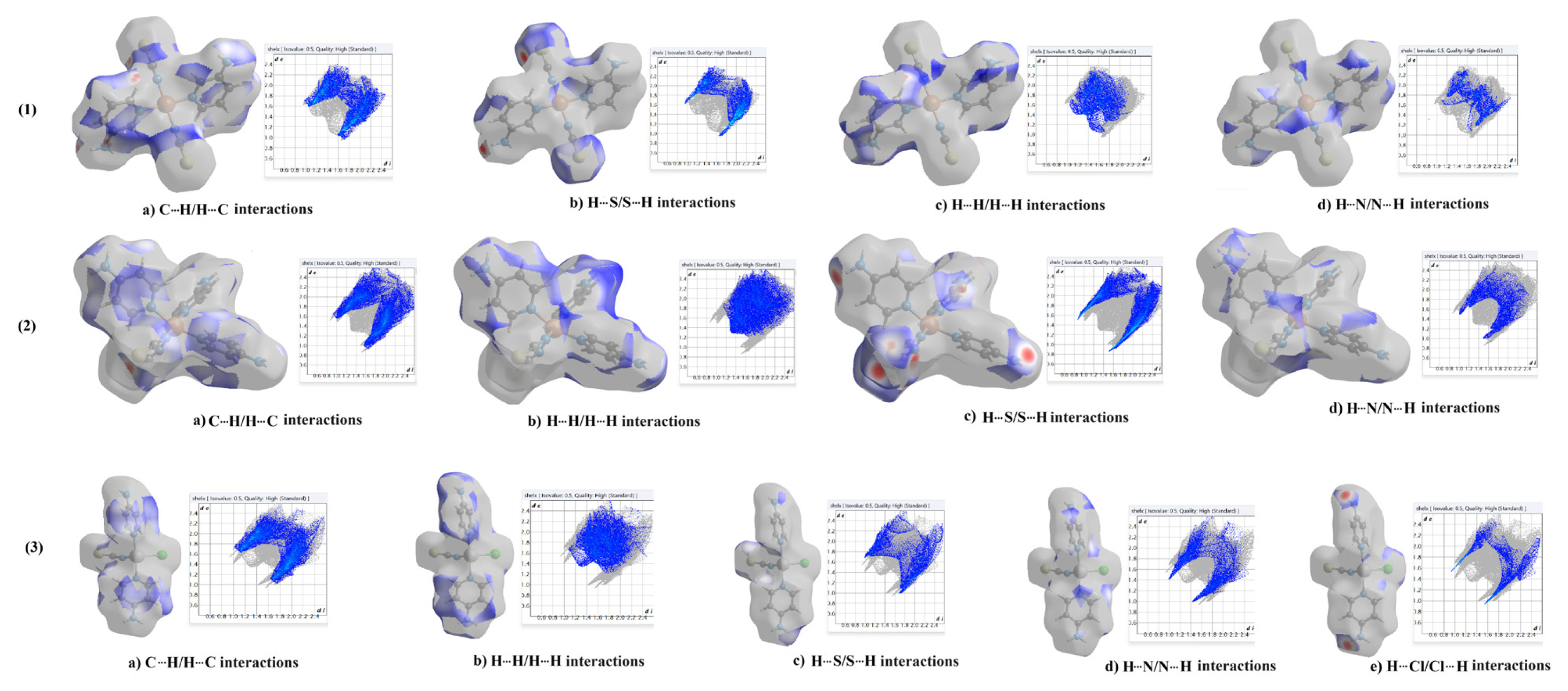

Supplement: Figure S2 — 2D fingerprint graphs obtained from the projections of some of the highest value intermolecular interactions of compounds 1, 2, and 3 on the dnorm Hirshfeld surface. [file tjc-48-05-780s2.tif]

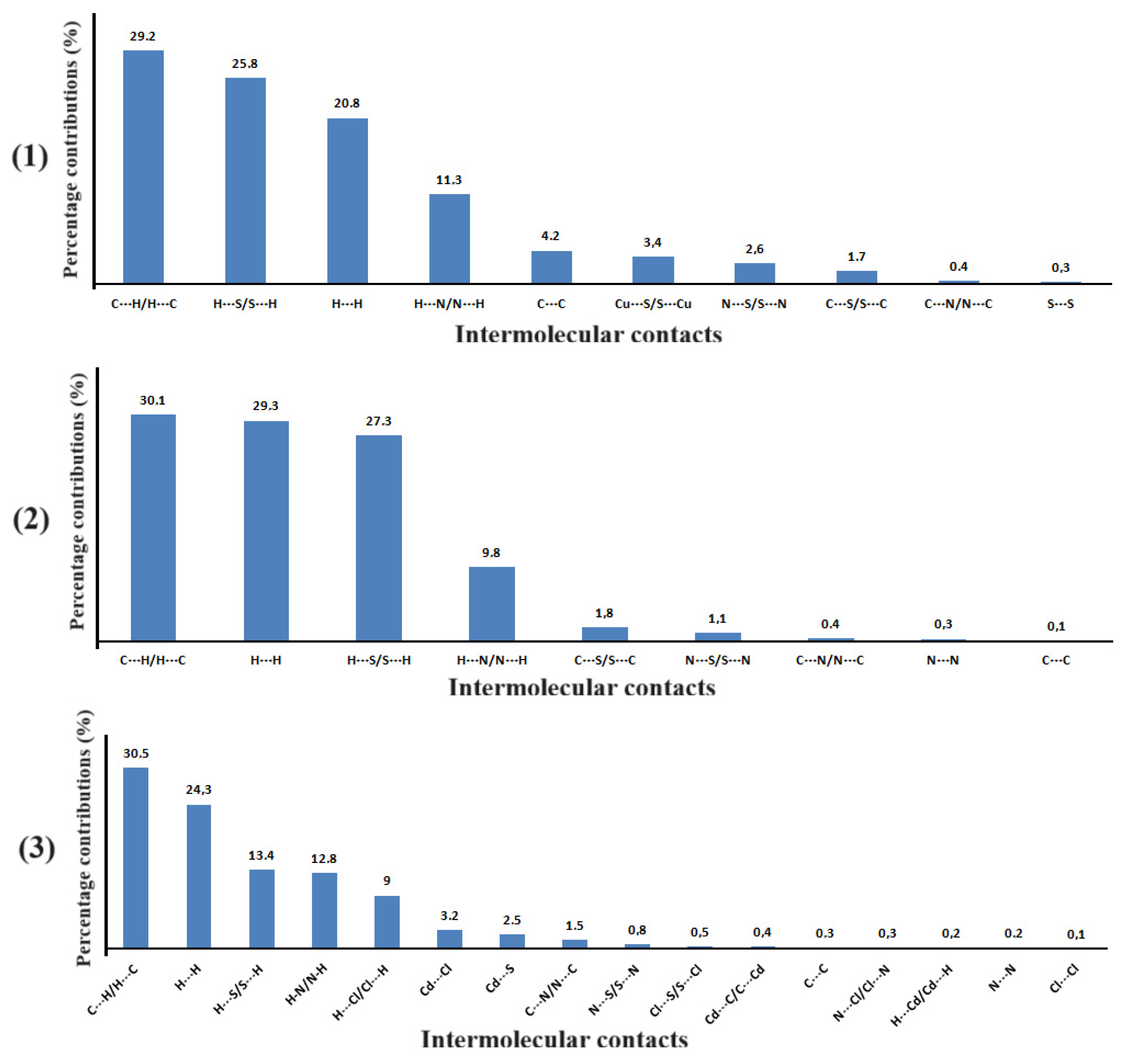

Supplement: Figure S3 — Percentage contributions of various intermolecular interactions of compounds 1, 2, and 3 in 2D fingerprint plots ordered from largest to smallest. [file tjc-48-05-780s3.tif]

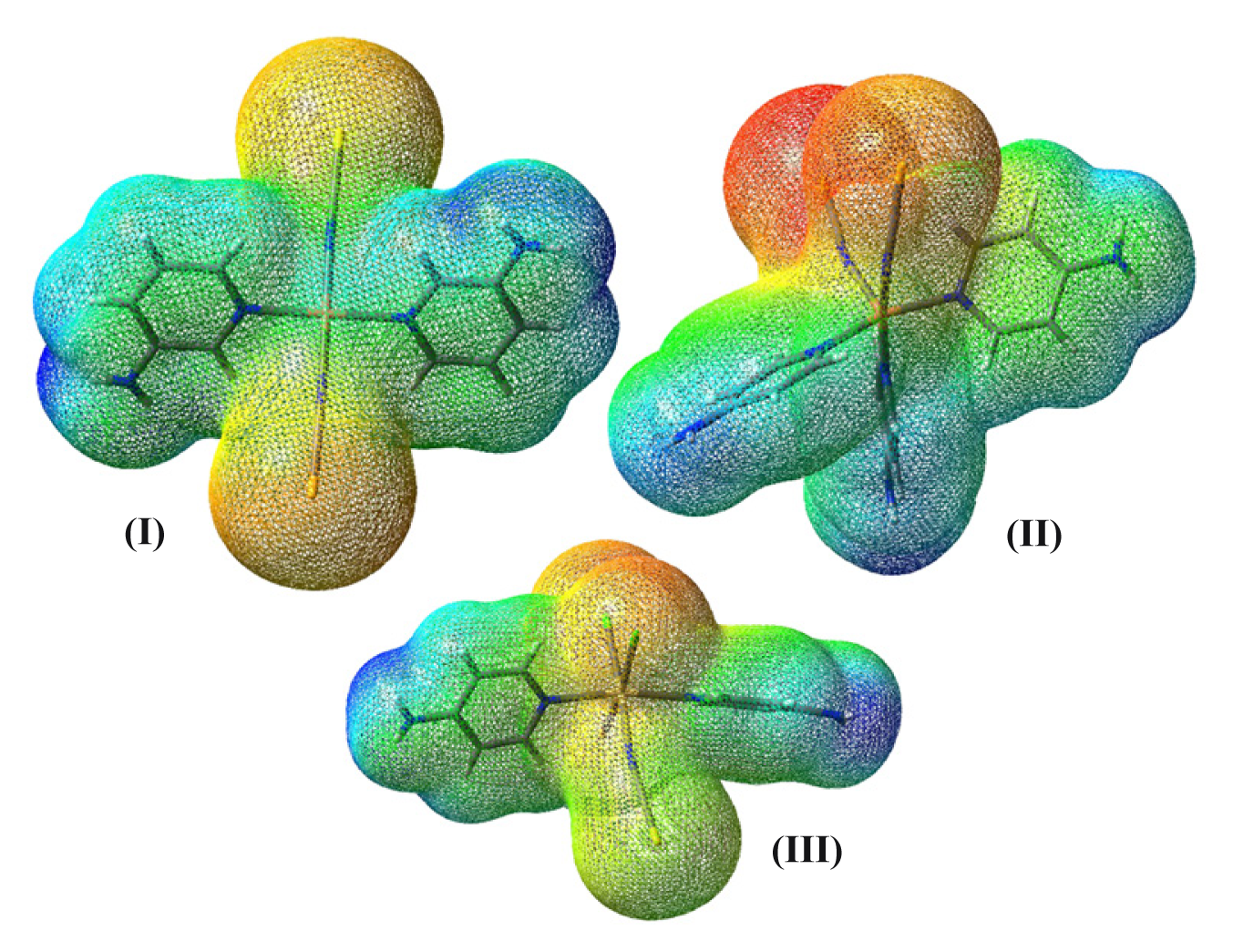

Supplement: Figure S4 — Molecular electrostatic potential maps of I, II, and III molecules calculated at DFT/B3LYP/LanL2DZ level. [file tjc-48-05-780s4.tif]

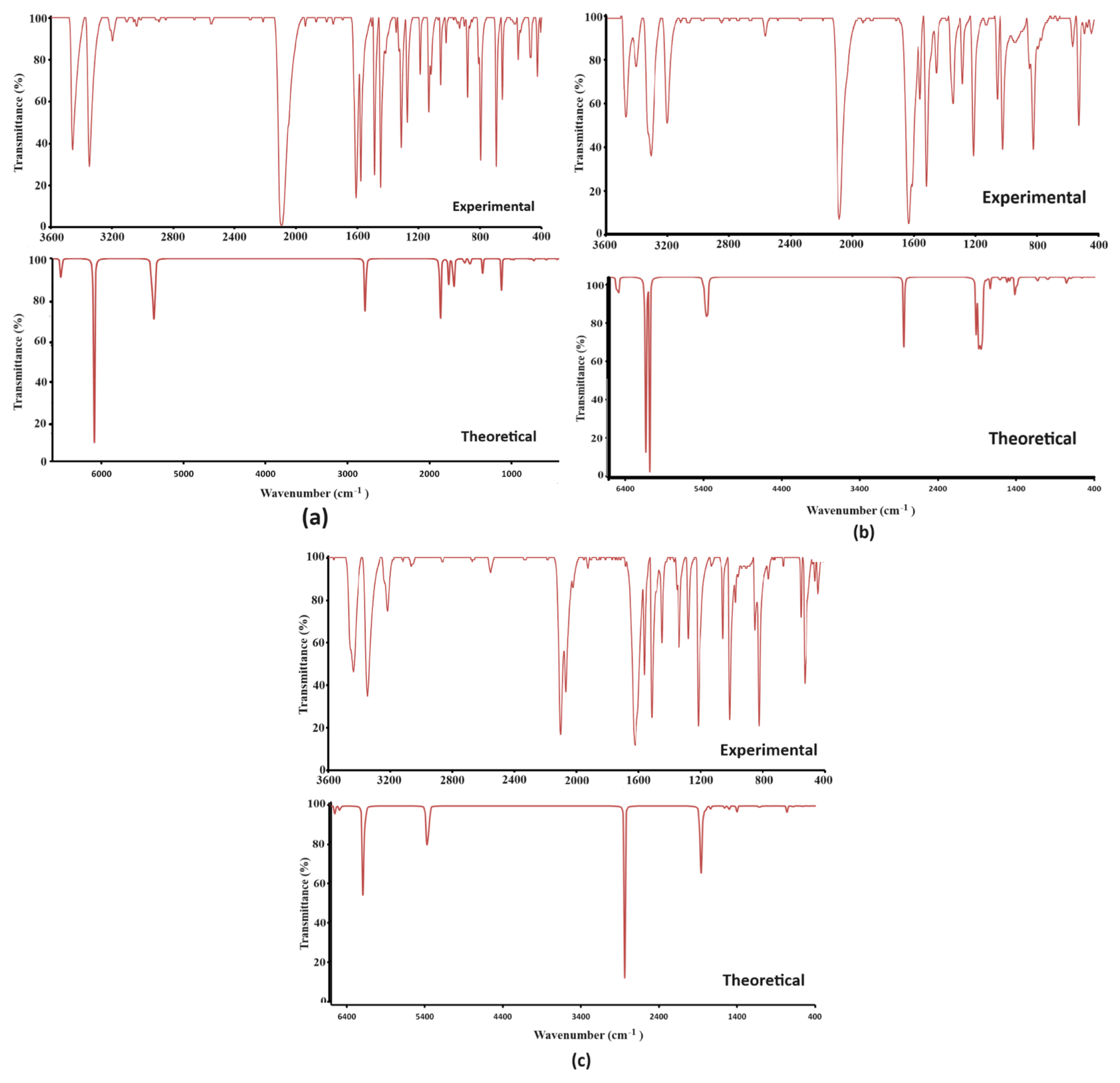

Supplement: Figure S5 — Experimental and theoretical FTIR spectra of compounds 1 (a), 2 (b), and 3 (c). [file tjc-48-05-780s5.tif]

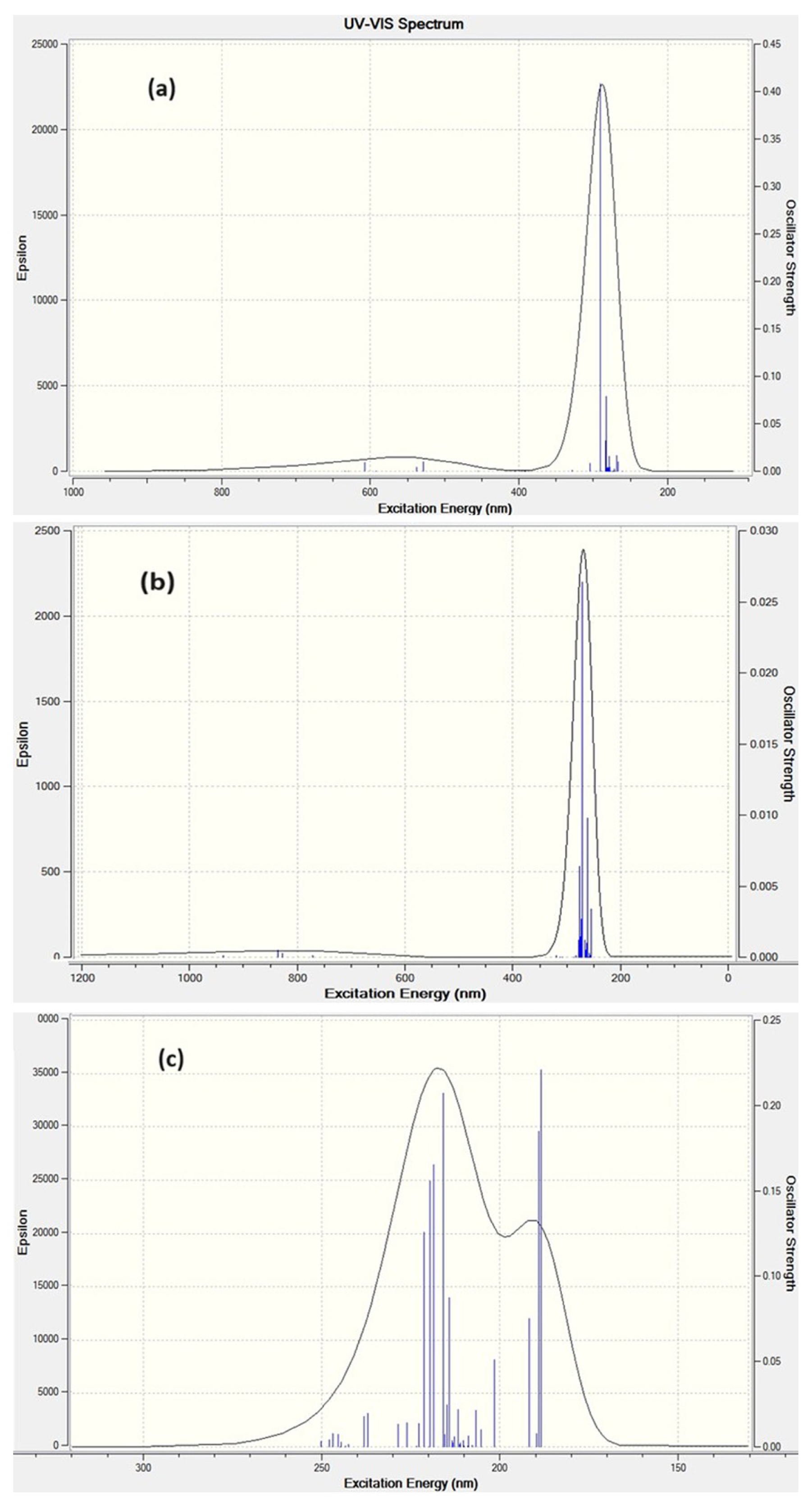

Supplement: Figure S6 — Theoretical UV–vis graphs of compounds 1 (a), 2 (b), and 3 (c). [file tjc-48-05-780s6.tif]

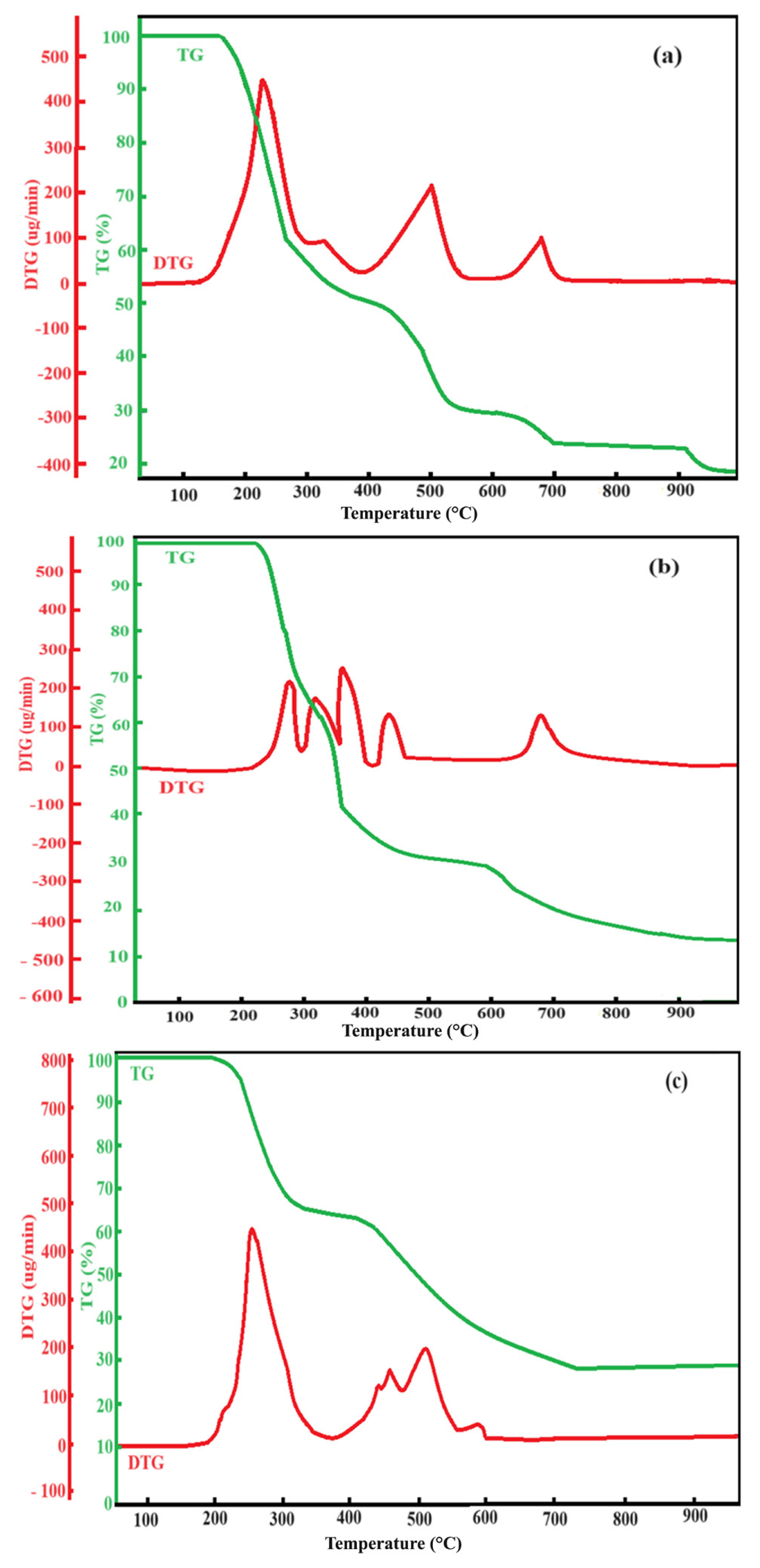

Supplement: Figure S7 — TGA and DTG curves of the thermal behavior of compounds 1 (a), 2 (b), and 3 (c). [file tjc-48-05-780s7.tif]
